# Biological Age in Congenital Heart Disease—Exploring the Ticking Clock

**DOI:** 10.3390/jcdd10120492

**Published:** 2023-12-10

**Authors:** Tijs K. Tournoy, Philip Moons, Bo Daelman, Julie De Backer

**Affiliations:** 1 Department of Cardiology, Ghent University Hospital, 9000 Ghent, Belgium; julie.debacker@ugent.be; 2 KU Leuven Department of Public Health and Primary Care, University of Leuven, 3000 Leuven, Belgium; 3 Institute of Health and Care Sciences, University of Gothenburg, 405 30 Gothenburg, Sweden; 4 Department of Pediatrics and Child Health, University of Cape Town, Cape Town 7700, South Africa; 5 Center for Medical Genetics, Ghent University Hospital, 9000 Ghent, Belgium

**Keywords:** congenital heart disease, aging, telomere length, epigenetic clock

## Abstract

Over the past 50 years, there has been a major shift in age distribution of patients with congenital heart disease (CHD) thanks to significant advancements in medical and surgical treatment. Patients with CHD are, however, never cured and face unique challenges throughout their lives. In this review, we discuss the growing data suggesting accelerated aging in this population. Adults with CHD are more often and at a younger age confronted with age-related cardiovascular complications such as heart failure, arrhythmia, and coronary artery disease. These can be related to the original birth defect, complications of correction, or any residual defects. In addition, and less deductively, more systemic age-related complications are seen earlier, such as renal dysfunction, lung disease, dementia, stroke, and cancer. The occurrence of these complications at a younger age makes it imperative to further map out the aging process in patients across the spectrum of CHD. We review potential feasible markers to determine biological age and provide an overview of the current data. We provide evidence for an unmet need to further examine the aging paradigm as this stresses the higher need for care and follow-up in this unique, newly aging population. We end by exploring potential approaches to improve lifespan care.

## 1. Introduction

Congenital heart disease (CHD) is the most common congenital defect, with an estimated birth prevalence of 8 cases per 1000 births [1,2]. It is defined as a gross structural abnormality of the heart or intrathoracic great vessels [3]. Over the past five decades, survival rates have significantly improved due to advancements in medical and surgical treatments. This has resulted in a major shift in the age distribution [4,5]. Where survival was limited, with only 49% reaching adult age in 1988 [5], to date, more than 95% of patients born with congenital heart disease will reach adult age [2,6], and at least 75% will reach 60 years [6]. With lifespan expanding, the focus is shifting from extending life expectancy towards improved quality of life and better options for participation in society, work, sports, and pregnancy. Despite great advances in diagnosis, treatment, and follow-up, the course of life differs from the ‘healthy adult’ with an excess of hospitalizations, interventions, and surgeries across all ages [7,8]. Adults with CHD are often well treated yet not cured where lifelong care and follow-up are required [7,9,10]. 

Resulting from the shift in age distribution, the number of adults now vastly exceeds that of children with CHD [7]. Interestingly, about a decade ago, the term ‘geriatric congenital heart disease’ was introduced [11]. Earlier, reaching older age was largely restricted to patients with simple heart lesions, whereas now even some adults with severe CHD are expected to survive into elderly age. Comparing elderly individuals with CHD to the general aging population is relevant since they face unique challenges and require specific follow-up and management [4,9,10,11,12]. One of the challenges is the rise of non-cardiac comorbidities, which presents complexities not only for clinical cardiologists. An illustrative example is Fontan Associated Liver Disease occurring in increasing numbers of young patients after Fontan correction. It is a severe complication and a major non-cardiac determinant of mortality. Guidance for diagnosis, follow-up, or treatment remains scarce, both for cardiologists and hepatologists [13,14,15]. 

It is important to note that the growing group of patients with CHD is very heterogeneous [1,4], with a wide spectrum of disease phenotypes, surgical and interventional procedures, and potential comorbidities and complications. Furthermore, the causes of CHD are diverse and remain largely unraveled. The developmental process of the heart is complex and influenced by the interaction of genetic, epigenetic, and environmental factors [16,17,18,19,20,21,22]. A clear genetic cause can be found in at most 20–35% of CHD cases. These encompass chromosomal aneuploidy such as Down syndrome and Turner syndrome, copy number variants such as 22q11 deletion syndrome and Williams–Beuren syndrome, single gene mutation syndromes like Alagille, Noonan, or CHARGE syndromes [20,21,22,23,24], and several monogenic non-syndromic lesions. Genetic entities are characterized by incomplete penetration and variable expression [23,25]. In addition to genetics, maternal obesity, diabetes, tobacco exposure, alcohol intake, teratogenic medications, and infections such as rubella are known causes [23,25]. The majority of disease presentations are non-syndromic with no distinct cause [18,21]. One of the other potential causal mechanisms for CHD is epigenetic factors which form a bridge between the genetic code and environmental modifiers. These epigenetic mechanisms are responsible for crucial regulation of the timely gene expression during heart development [17,24,25]. It has been postulated that the timed expression, other than the gene product itself, is important in the pathophysiology of CHD [25]. 

## 2. Lifespan Research in CHD

### 2.1. Aging Research

Aging is a fundamental biological process affecting all human beings [26,27]. With advancing human life expectancy, there has been growing attention to delaying the natural decline of physiological integrity. This decline is a result of accumulating molecular and cellular damage over time [27,28,29]. While aging is a universal process, the pace can vary significantly among individuals. Accelerated aging occurs when an individual’s biological age exceeds what would be expected based on their chronological or calendar age. This is clinically manifested by the early onset of age-related diseases and conditions or the deterioration of physiological functions [29]. An extreme form of accelerated aging is seen in rare genetic conditions like Hutchinson–Gilford progeria syndrome and Werner syndrome [28]. These patients show caricatural clinical features of early, rapidly escalating physiological aging such as hair loss, skin atrophy, and joint stiffness. Furthermore, age-related complications such as myocardial infarction, heart failure, stroke, and malignant tumors occur before reaching the age of 20 years [30]. Another, subtler example of progeroid presentation is observed in patients with Down syndrome. In these patients, accelerated aging is segmental and mainly present in endocrine and immune functions [31,32,33,34]. Other than genetic causes, exposure to traumatic stress has been identified as an accelerator of the aging pace [35,36,37,38]. This implies that adverse experiences during early life, physical or psychological, have the potential to impact the aging trajectory.

### 2.2. Indications of Accelerated Aging in CHD

**Figure 1:**
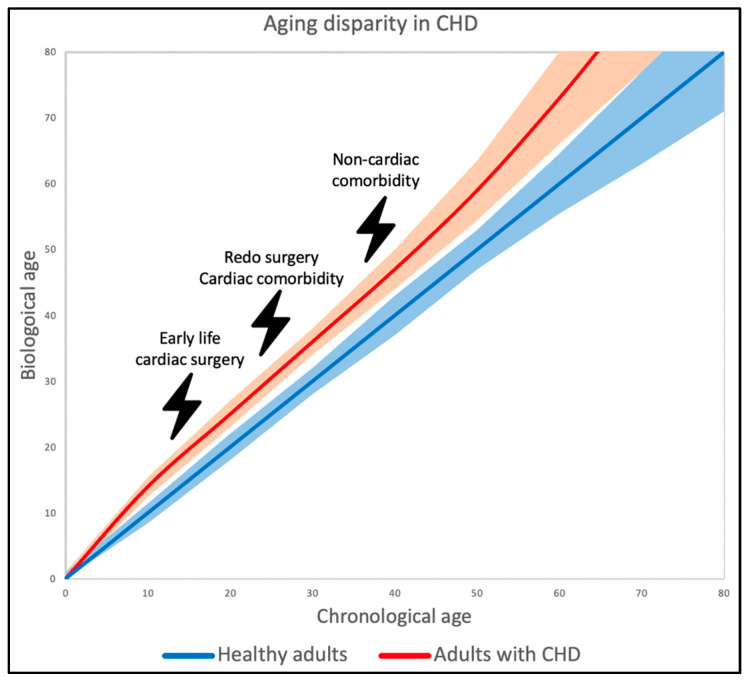
Age disparity between healthy adults and adults with CHD. In healthy adults, it is expected that chronological and biological age are roughly identical. A higher pace of biological aging is expected in adults with CHD as is illustrated by the progressive increase of biological age.

The newly aging group of patients with CHD has come into the spotlight. Namely, the impact of imperfect hemodynamics as well as early life traumas resulting from often repeated early life surgeries and hospitalizations on the lifespan of these patients needs to be assessed [9,11,39]. Data indeed suggest a higher pace of aging in patients with CHD [9,39]. This is illustrated in Figure 1, showing that surgery and residual lesions impact the aging dynamics thus accelerating the development of age-related comorbidities.

Early mortality can be a sign of accelerated aging. In CHD, life expectancy is improving yet still reduced with a mortality excess across all ages [40]. Mortality is more than three times higher compared to healthy adults [6] with the highest risk in complex CHD such as Fontan circulation and Eisenmenger syndrome. Early mortality is related to surgical procedures and potential complications which are not attributable to aging dynamics. Later in life, non-cardiac related causes of mortality become more prominent [7,11,40]. Cancer, infections, pulmonary disease, and neurological disorders emerge as important causes of death [4,41]. It is postulated that mortality moves beyond the congenital heart defect in adult age and is a consequence of general accelerated aging processes [11,42].

### 2.3. Young Patients with Old Hearts

The earlier occurrence of age-related diseases is another argument for an accelerated aging process. As individuals with CHD grow older, they experience physiological changes with an increased risk of cardiovascular complications [7,9]. In the general population, heart failure, arrhythmia, stroke, and coronary artery disease become increasingly prevalent with advancing age. In CHD, epidemiological data consistently show an excess of these cardiovascular disease manifestations, albeit with significant variation in effect sizes [43,44]. Aging is not the main driver of disease in CHD when taking into account the impact of the original defect or its (incomplete or palliative) correction, residual flow obstruction, valvular dysfunction, shunts, scarring, myocardial ischemia, pressure/volume overload, etc. [44,45]. These mechanisms contribute to heart failure, which is the main cause of morbidity with a prevalence of 20–50% of adults with CHD [1]. Particularly in complex CHD, the risk of heart failure is already present at a young age [4,46] and is also the leading cause of mortality [40,47]. Despite the major burden, only small heart failure drug trials are available in adults with CHD with conflicting results. Treatment strategies are mainly extrapolated from the ‘acquired’ heart failure guidelines. This may not necessarily be the most adequate treatment, since underlying mechanisms in CHD differ from the acquired heart failure that occurs in older adults without CHD. This is particularly the case in systemic right ventricle failure or a failing Fontan circulation [4,45,46,47,48]. Both atrial and ventricular arrhythmia are common complications. Atrial arrhythmia was shown to occur in 15% of adults with CHD [49]. Arrhythmogenic driving mechanisms are ischemia, scarring, due to earlier interventions, and remodeling, due to chronic pressure/volume overload. This can cause significant morbidity and sudden cardiac death [40,50]. The type of arrhythmia and the potential clinical implications vary across the spectrum of CHD. Patients with tetralogy of Fallot and transposition of the great arteries after atrial switch procedure are especially at risk. Heart failure and arrhythmias interact synergistically and are enhanced by each other’s underlying mechanisms [51]. In children and young adults with CHD, the risk of ischemic stroke is more than 10 times higher when compared to matched healthy peers [52]. The disparity in the prevalence of ischemic stroke is highest in younger adults. However, this remains significant across adulthood and beyond 60 years. Common risk factors such as arrhythmia, heart failure, and infective endocarditis are more prevalent in CHD. Residual shunts causing paradoxical embolisms, residual structural abnormalities, and iatrogenic complications are potential pathophysiological mechanisms [49,52,53]. The absolute risk of coronary artery disease (CAD) across the spectrum of CHD is generally comparable to healthy adults [54]. Significant CAD was found in 9.2% when performing coronary angiography in adults with mainly mild- and moderate CHD. Cyanotic CHD is relatively protective [55], whereas patients with coarctation of the aorta have an elevated risk of CAD, which is mediated by the high prevalence of arterial hypertension [56]. The risk of myocardial infarction is twice as high when compared to healthy counterparts, and despite having fewer comorbidities due to the younger age of onset, patients with CHD have a worse outcome [57,58]. Additional potential contributing factors are anomalous coronary anatomy, coronary manipulation during surgery, or paradoxical embolisms through residual shunts causing coronary obstruction [44,57]. 

As already mentioned, the underlying cardiac defect impacts the increased risk of accelerated (cardiovascular) aging manifestations substantially but other factors must also be taken into account. Conventional cardiovascular risk factors for example have been documented with increased prevalence in the CHD population [58,59,60,61]. In the study of Moons et al., 80% of young adults with CHD (mean age 26 years) had at least one risk factor for CAD such as smoking, sedentarism, hypertension, or obesity [60]. This is alarming and might even be an underestimation, especially since dyslipidemia was not incorporated in this study, while it is known to be more prevalent in adults with CHD [58,61]. Tobacco use in adults with CHD (12–19%) is lower than in the healthy population (25–30%), yet the number is still worrisome [60,61,62]. The link between type 2 diabetes, another cardiovascular risk factor, and CHD is more disputed with the risk being highest in cyanotic CHD patients [60,61,63]. One could argue that the presence of major cardiovascular risk factors at a young age following the trajectory caused by the congenital heart defect impacts the further lifespan with the development of different cardiovascular comorbidities. 

### 2.4. CHD beyond Old Hearts

The aforementioned observations led to the notion of “young patients with old hearts”. It can, however, be assumed that manifestations extend beyond the heart and that “young patients with old bodies” would be a more appropriate wording. Namely, other organ dysfunction commonly acknowledged as age related are more prevalent and occur at a younger age in CHD [43,59]. In some patients, extra-cardiac manifestations can be linked to an underlying (genetic) syndrome. However, as most cases of CHD are non-syndromic, this observation is surprising and intriguing. 

For example, reduced pulmonary function is present in nearly 50% and associated with higher mortality. Restrictive lung disease is the most frequently occurring pattern, especially in Fontan circulation and Tetralogy of Fallot. This is often related to previous thoracotomies, diaphragmatic palsy, cardiomegaly, scoliosis, or early life pulmonary hypoperfusion [64]. Renal function is known to decrease with age. However, pathologic renal dysfunction is often present in patients with both simple and complex CHD lesions. Very illustrative is a comparative study which showed mild renal dysfunction in 41%, and moderate or severe impairment in 9% of adults with CHD (mean age 36 ± 14.2) [65]. 

In a study looking at musculoskeletal features, almost half of the adults with complex CHD (mean age 35.8 ± 14.3) were categorized as sarcopenic [66]. In the same study population, a significantly lower bone mineral density was seen, illustrating an excess of early onset osteoporosis [67]. Another common manifestation of aging is neurocognitive decline and the development of dementia. Since the geriatric CHD population is relatively new, data on dementia are limited and prone to survival bias. The neurodevelopmental processes seem already disturbed at young age in patients with CHD due to genetic abnormalities, cerebral hypoxia, or stroke. Acquired cardiovascular comorbidities on top of the congenital defect might accelerate the neurocognitive decline [68]. Only one cohort study looked at the incidence of dementia. The hazard ratio for ‘early onset’ dementia (<65 years) was 2.6 (95%CI 1.8–3.8) when comparing CHD to the general population. In older patients (>80 years), the incidence was rather similar, which can be attributed to the excess of simple CHD with survival bias [69]. The prevalence of cancer in CHD is roughly two times higher in children, adolescents, and adults [70,71]. This is often attributed to radiation exposure, which largely occurs during childhood with less matured organs being more sensitive to develop malignancies [72,73]. The distribution of cancer types is roughly the same as in the general population with a relative excess of leukemia [70]. CHD is proposed to be a group at risk for malignancies thus warranting earlier screening. The risk is higher in more recent birth cohorts, which reflects the improved survival of patients with more complex CHD who live long enough to acquire malignancies [71,74], as the prevalence is highest in complex CHD [73]. The impact of early life exposure to radiation in the development of cancer is well established, specifically in adults with CHD when compared with matched controls [73,74]. Controversially, the elevated cancer risk was found regardless of any previous surgical procedures with inherent radiation exposure compared to the general population [71]. This might illustrate other potential contributing factors in this specific population, such as genetic conditions. For example, patients with Down syndrome have a 10–20 times higher risk of leukemia [70,73]. Furthermore, some in utero toxins are both teratogenic with an effect on cardiac development and carcinogenic [70]. 

**Figure 2:**
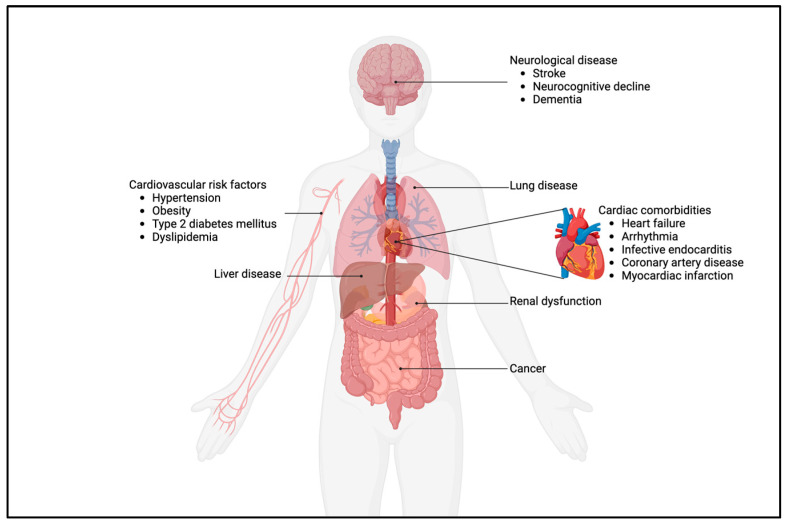
Cardiac and non-cardiac comorbidities in adults with CHD.

Thus, there is an excess of both cardiac and non-cardiac comorbidities, as illustrated in Figure 2. In adults with CHD, these are consistently more prevalent and occur earlier when compared to the general population. Therefore, one could pose that the impact of the early life trajectory caused by the heart defect moves beyond the heart, especially in complex CHD. Interestingly, patients with mild CHD, such as patent ductus arteriosus (PDA), isolated atrial septum defect (ASD), ventricular septum defect (VSD), and pulmonary stenosis, are not exempted from aforementioned comorbidities like stroke, pulmonary disease, heart failure, myocardial infarction, and neurological disease, as was shown by a recent Danish population study [42]. This further underscores that adequate care—even in mild CHD—does not exempt patients from elevated healthcare needs, thus further warranting lifelong follow-up in all CHD. 

## 3. Measuring Biological Age in CHD 

When assessing the unique health challenges faced by individuals with CHD, it is clear that factors such as the congenital heart defect itself, the (multiple) surgeries and interventions, the early life exposure to radiation, and the often incomplete correction of congenital heart lesions could collectively subject the patient to a considerable degree of physical, metabolic, and psychological stress [39]. All these stressors, mainly encountered in early life, have the potential to induce premature aging. As a result, it is imperative to recognize that comparing the various physiological functions of adults with CHD directly to those of ‘healthy adults’ may not provide an accurate reflection of their true health status [9,10]. To better appreciate the nuanced differences in health states, it is important to define objective biological markers that offer a deeper understanding of this health status. One such marker gaining increasing attention is the concept of biological age, which can differ from a person’s chronological age or calendar age. Biological age integrates the chronological age with the individual’s physiological state, based on genetics, lifestyle, comorbidities, and overall health [29].

The observation of more age-related complications at a younger age, as described above, raised the hypothesis of an elevated biological age in patients with CHD [9,39]. A higher biological age than chronological age is also known as accelerated aging. Understanding the concept of biological age and its relevance in assessing the aging process among individuals with CHD is crucial for optimizing their care and improving long-term outcomes [9]. Consequently, utilizing biological age as a metric offers a more comprehensive and nuanced understanding of the health status and needs of adults with CHD.

To date, there is no gold standard for measuring biological age since the aging process is complex with many contributing factors. In 2013, nine hallmarks of aging were described by López-Otín et al. These affect the aging process, are interconnected, and consist of telomere attrition, epigenetic alterations, genomic instability, cellular senescence, stem cell exhaustion, loss of proteostasis, deregulated nutrient-sensing, mitochondrial dysfunction, and altered intercellular communication [27]. An update of these was recently made with the addition of disabled macroautophagy, dysbiosis, and chronic inflammation [75]. Numerous candidate markers for biological age estimation exist, including biomarkers and functional parameters, each with their own advantages and disadvantages. Common candidates are telomere length, the epigenetic clock, serum markers, and frailty [29,39,76,77,78]. Hereafter, we summarize markers with relevance for adults with CHD.

### 3.1. Telomere Length 

A first option for biological age estimation is the telomere length (TL) since it is appreciated as one of the ‘hallmarks of aging’ [27,75]. Telomeres are the protective end caps of chromosomes and consist of repetitive hexametric nucleotides (TTTAGG). Their function is to prevent loss of DNA during cell replication and avoid chromosome-fusion. Across all tissues, telomere shortening, or ‘attrition’ is caused by repeated replication and affected by oxidative stress and inflammation [79,80,81,82]. When reaching a critical length, cell senescence follows. This is known as the Hayflick limit and illustrates why telomeres are sometimes referred to as a ‘mitotic clock’ [79,80,81]. The introduced senescence is protective since it halts cell division in cells with diminished DNA integrity thus preventing malignant conversion [28,83]. The protective role of telomere-attrition and cell senescence is illustrated by the paradoxical observation of a higher risk of neoplasms in patients with mutations in POT-1, which is an important protein in telomere maintenance and results in significantly longer telomeres [84]. 

Since telomeres shorten with age, TL can be used as a biological age parameter [79,80,81,82,85]. When measuring a patient’s TL, leukocytes from whole blood are typically used. They are the most pragmatic choice and can be used as a proxy marker for the TL in most other tissue types [85,86]. It is important to note that different tissues have varying telomere length due to their distinct rates of cell proliferation [86]. It is argued that shorter telomeres are not only a marker of aging but have a causative role in the development of age-related diseases since telomerase expression, which maintains TL, could delay their onset [82]. Individual TL is associated with parental TL and affected by smoking status, diet, socioeconomic status, physical activity, and stress levels [79,82,85,87]. Shorter TL is proposed to be associated with overall mortality and age-related diseases. For example, shorter telomeres are associated with type 2 diabetes mellitus, myocardial infarction, heart failure, cognitive impairment, stroke, and some cancers [79,80,81,82,85,88,89,90]. There are different methods available for quantifying TL [89]. In epidemiological settings, quantitative polymerase chain reaction (qPCR) techniques are first choice. qPCR measures average TL, illustrating the attrition process but is prone to substantial between and within batch variance [91]. TL is affected by both genetic and non-genetic factors and can therefore be used as an interactive factor representing both the inherited length as well as the impact of different stressors faced during the lifetime [81].

**Figure 3:**
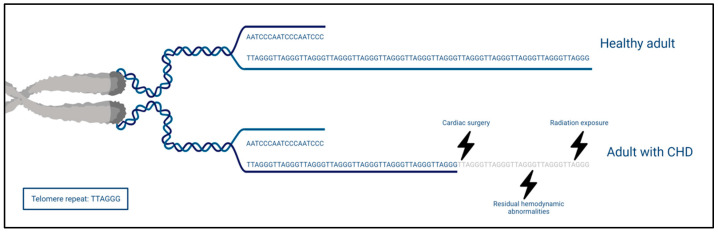
Telomere length in healthy adults and adults with CHD. The telomeres, consisting of repetitive hexametric nucleotides (TTAGGG), are simplified in their depiction (based on Calado et al. [79]). The telomeres of adults with CHD are shortened due to different factors such as surgery, radiation exposure, and residual hemodynamic abnormalities.

Despite the general interest in TL, only one small cross-sectional study of 50 adults with CHD (age 25.2 ± 9 years) has examined the leukocyte telomere length (LTL) [92]. Significantly shorter telomeres (23%) were observed when compared with age- and sex-matched healthy controls. Additionally, an inverse correlation was found between LTL and cumulative radiation exposure caused by X-rays, computed tomography, and catheterizations. The authors attribute the observation of shorter LTL to the radiation exposure but admit they cannot completely rule out other stressors which might impact LTL. Cumulative radiation in children, who are more vulnerable to the effect of radiation, is potentially important in the further course of life as it is associated with cancer risk [72,93,94]. Interestingly, the shorter TL is an additional pathway for the observation of more malignancies [73], as we mentioned earlier in the systemic comorbidities of CHD. We argue that, other than radiation exposure, the congenital heart lesion itself, ensuing imperfect biomechanics that may result in oxidative stress and low-grade inflammation, accelerates biological aging and telomere shortening (Figure 3). However, one small cross-sectional study is insufficient to make such assumptions. 

### 3.2. Epigenetic Clock 

Another hallmark of aging are epigenetic alterations, the main mechanisms being DNA methylation, histone modification, and non-coding RNAs [27]. These are reversible processes which can influence gene expression without changing the genetic code. Epigenetics are seen as the interplay between genetics and environmental influences. DNA methylation is the addition of methyl groups to the cytosine base of DNA in a cytosine- guanine dinucleotide (CpG). Some CpGs are clustered in dense areas named CpG-islands. A global loss of methylation is seen with advancing age. In some CpG islands, hypermethylation is seen. Using a machine learning approach on the methylation status of specific CpG islands resulted in an unexpected level of accuracy of age prediction [76,77,95]. Following this, Horvath et al. developed the first pan-tissue epigenetic ‘clock’ in 2013 [95]. Since then, other clocks have been developed. Frequently used examples are Hannum’s clock [96], Levine’s PhenoAge [97], and GrimAGE [98] which are based on different CpG islands or integrating other clinical and biochemical parameters. Accelerated ticking of the epigenetic clock is a predictor of mortality [99]. A higher pace of ticking is seen in different conditions such as diabetes, cardiovascular disease, stroke, HIV infection, chronic obstructive lung disease (COPD), Parkinson’s disease, and (risk of) cancer [72,76,77,100,101,102,103,104,105,106,107]. The extent of the associations varies between different epigenetic clocks [100]. This suggests that each clock might be influenced by different aspects of the complex aging process. It is unclear whether aging-related methylation changes are consequence or cause of aging. In the former hypothesis, epigenetic age is a promising predictor of biological age. In the latter hypothesis, it might also be a suitable target for anti-aging strategies [76,77]. 

In recent years, the main application of epigenetics in CHD has been to further unravel the disease pathophysiology and etiology as this remains elusive in a large proportion of patients. Differential methylation patterns have been described in CHD, both generally across the genome and in specific genes [16,18,19,24,108]. The knowledge and impact of aberrant methylation patterns is still evolving [18,44]. However, it would be shortsighted to restrict its role to the developmental phase alone. The use of epigenetic clocks illustrates that methylation changes are also representative of a change in the ticking rate of the biological clock [39,77]. To the best of our knowledge, no studies have been conducted to determine whether accelerated epigenetic age is present in patients with CHD. As illustrated above, with higher incidence of age-related comorbidities as well as higher mortality throughout all ages, it is conceivable that a higher epigenetic age could be observed and an association with disease severity might exist. This hypothesis is further backed by the fact that epigenetic clocks tick faster in childhood and that important life events, like CHD surgery, would have a greater impact than acquired heart diseases at adult age [35,107].

### 3.3. Inflammaging 

Telomere length and epigenetic clock are both to date the aging biomarkers that have been studied and validated the most [76]. In recent years, growing attention has gone to the role of low-grade inflammation in the development of different age-related comorbidities, called inflammaging [109]. Inflammation was not recognized as one of the original nine hallmarks of aging but has recently been added as an ‘integrative hallmark’ which arises when damage cannot be compensated [75,110]. The presence of high level pro-inflammatory cytokines is associated with cardiovascular disease, cancer, chronic kidney disease, and cognitive impairment [111]. It is argued that the aforementioned age-related diseases are not only the result of this sterile inflammation but that these comorbidities in turn accelerate the overall aging process creating a vicious cycle [109]. 

There is growing evidence of an activated immune response in patients with CHD, following impaired hemodynamics, scarring due to surgery and hypoxemia [112,113], and the often iatrogenic (partial) removal of the thymus during open heart surgery [113]. The exact interaction between the immune system and CHD remains elusive. High sensitivity C-reactive protein (hsCRP), a pro-inflammatory marker which reflects a chronic inflammatory state is elevated in 25–28% of patients with CHD. This observation favors a potential inflammatory link in CHD. Furthermore, elevated hsCRP is associated with heart failure, arrhythmia, thromboembolic events, and all-cause mortality [114,115]. Independently of CRP, elevation of red cell distribution width is also associated with cardiovascular events [116]. Prospective studies showed elevated tumor necrosis factor α and interleukin-6 in young adults and children with CHD. This was also related to functional status [117,118]. These findings suggest a chronic low-grade inflammatory state in patients with CHD which might be an additional driving mechanism for accelerated aging and the early occurrence of age-related comorbidities [113]. In contrast to DNA-markers, such as TL and epigenetic age, these markers are more feasible to use in clinical care. However, to date, there are insufficient data to implement these.

### 3.4. Frailty & Functional Parameters

Aging is often associated with a decrease in functional reserve, resistance, and resilience of multiple organ systems, which may be approximated as frailty. This makes the individual vulnerable, leading to a higher risk of accelerated functional decline and adverse health-related outcomes [119]. Frailty is therefore associated with an increased risk of falling, disability, hospitalization, institutionalization, unmet healthcare needs, lower quality of life, and mortality [120]. Furthermore, it is an important parameter in understanding the aging process. There are multiple ways to operationalize frailty. The frailty phenotype by Fried et al. [121] and the complementary Frailty index by Rockwood et al. are the most frequently used methods [122]. 

To date, frailty has not been comprehensively explored in patients with CHD. Only preliminary data from the APPROACH-IS II (Assessment of Patterns of Patient-Reported Outcomes in Adults with Congenital Heart disease–International Study 2) project is available [123]. In this study, the presence of frailty in patients with moderate or complex CHD, aged 40 years or older, was evaluated using the Fried method. Initial analyses of 79 patients revealed that 41.8% were at risk for frailty and 8.9% were classified as frail [9]. Since the geriatric CHD population is emerging, frailty assessment is of relevance due to the potential impact on overall health.

## 4. Avenues for Future Research 

Despite the continuously growing data suggesting an accelerated aging pattern across the spectrum of patients with CHD, biological aging markers have only been studied scarcely [9,39]. Considering the impact of CHD on a patient’s lifespan, we argue that an in-depth investigation across all ages of telomere length, epigenetic clock, frailty, and inflammation markers would be informative. By exploring telomere and epigenetic dynamics across the ages, we move beyond genetics as merely a static code but approach it as a dynamic structure which could provide important clues of the lifespan course of patients with CHD. An age-stratified group comparison of patients across the lifetime of patients with CHD could investigate the pace of aging and the impact of both early life surgery and late onset complications (Figure 1). Additionally, it is of interest to assess whether the severity of CHD impacts the aging dynamics. Practical issues such as between batch variances in the DNA-analyses are noteworthy potential limiting factors that need to be considered [124].

The correlation between telomere length and epigenetic clock is rather low [29,76,124] which is an illustration of the complexity of the aging process where each marker highlights a fraction of the aging process with different aging hallmarks interacting with each other. For example, it is postulated that telomeres have an anti-inflammatory function. Following this, longer telomere length not only protects cells from senescence but also delays the inflammaging pathway [125]. Furthermore, DNA markers like TL and epigenetic clock are based on one tissue type which is used as proxy for the whole individual and might represent all aging factors [17,126]. Therefore, it is important to look beyond one marker to achieve a more holistic view [124]. Efforts have been made to develop composite biomarkers which integrate different pathways and give a more robust perspective [127]. In the absence of a gold standard, looking beyond one parameter is relevant, thus integrating different aging pathways in order to create a general view of the age-related changes. 

## 5. Conclusions

The increasing life expectancy of adults with CHD is a major accomplishment of modern medicine. The focus of care has gone beyond survival towards a healthy lifespan. Adults with CHD are not cured but remain vulnerable to both cardiovascular and non-cardiac age-related diseases at a younger age compared to the general population. Some of them are deductive from the early life trajectory, yet this might not entirely account for the high disease burden. We therefore want to stress the high need for an in-depth investigation of the biological aging processes by examining telomere length, epigenetic clocks, and functional parameters across the ages and spectrum of CHD. This knowledge could guide the development of precision medicine, tailored screening and interventions, improved risk stratification strategies, and more personalized care plans for this population. Exploring the biological age across the lifespan acknowledges that the effect of CHD is not solely confined to the structural heart lesion but also affects various physiological and cellular processes throughout the body which may accelerate the overall aging process. 

## Data Availability

No new data were created or analyzed in this study. Data sharing is not applicable to this article.
